# Association between tumor ^18^F-fluorodeoxyglucose metabolism and survival in women with estrogen receptor-positive*,* HER2-negative breast cancer

**DOI:** 10.1038/s41598-022-11603-z

**Published:** 2022-05-12

**Authors:** Sun Young Chae, Seol Hoon Park, Hyo Sang Lee, Jin-Hee Ahn, Sung-Bae Kim, Kyung Hae Jung, Jeong Eun Kim, Sei Hyun Ahn, Byung Ho Son, Jong Won Lee, Beom Seok Ko, Hee Jeong Kim, Gyungyub Gong, Jungsu S. Oh, Seo Young Park, Dae Hyuk Moon

**Affiliations:** 1 Department of Nuclear Medicine, Uijeongbu Eulji Medical Center, Eulji University School of Medicine, Uijeongbu-si, Republic of Korea; 2 Department of Nuclear Medicine, Ulsan University Hospital, University of Ulsan College of Medicine, Ulsan, Republic of Korea; 3 Department of Nuclear Medicine, GangNeung Asan Hospital, University of Ulsan College of Medicine, Gangneung, Republic of Korea; 4 Department of Oncology, Asan Medical Center, University of Ulsan College of Medicine, Seoul, Republic of Korea; 5 Department of Surgery, Asan Medical Center, University of Ulsan College of Medicine, Seoul, Republic of Korea; 6 Department of Pathology, Asan Medical Center, University of Ulsan College of Medicine, Seoul, Republic of Korea; 7 Department of Nuclear Medicine, Asan Medical Center, University of Ulsan College of Medicine, 88, Olympic-ro 43-gil, Songpa-gu, Seoul, 05505 Republic of Korea; 8 Department of Clinical Epidemiology and Biostatistics, Asan Medical Center, University of Ulsan College of Medicine, Seoul, Republic of Korea; 9 Present Address: Department of Statistics and Data Science, Korea National Open University, Seoul, Republic of Korea

**Keywords:** Biomarkers, Oncology

## Abstract

We examined whether ^18^F-fluorodeoxyglucose metabolism is associated with distant relapse-free survival (DRFS) and overall survival (OS) in women with estrogen receptor (ER)-positive, HER2-negative breast cancer. This was a cohort study examining the risk factors for survival that had occurred at the start of the study. A cohort from Asan Medical Center, Korea, recruited between November 2007 and December 2014, was included. Patients received anthracycline-based neoadjuvant chemotherapy. The maximum standardized uptake value (SUV) of ^18^F-fluorodeoxyglucose positron emission tomography/computed tomography (PET/CT) was measured. The analysis included 466 women. The median (interquartile range) follow-up period without distant metastasis or death was 6.2 (5.3–7.6) years. Multivariable analysis of hazard ratio (95% confidence interval [CI]) showed that the middle and high tertiles of SUV were prognostic for DRFS (2.93, 95% CI 1.62–5.30; *P* < 0.001) and OS (4.87, 95% CI 1.94–12.26; *P* < 0.001). The 8-year DRFS rates were 90.7% (95% CI 85.5–96.1%) for those in the low tertile of maximum SUV vs. 73.7% (95% CI 68.0–79.8%) for those in the middle and high tertiles of maximum SUV. ^18^F-fluorodeoxyglucose PET/CT may assess the risk of distant metastasis and death in ER-positive, HER2-negative patients.

## Introduction

Estrogen receptor (ER)-positive, HER2-negative breast cancer accounts for 60–70% of breast cancers^1^. Although patients are treated with curative intent, > 20% of patients experience relapse within 10–15 years and die from metastatic disease^2^. The recommended systemic treatment for patients with operable but high-risk tumors and those with locally advanced disease includes chemotherapy, followed by endocrine therapy. However, the diverse molecular characteristics of patients lead to diversity in the response to systemic therapies^3,4^. ER-positive, HER2-negative breast cancer does not benefit from systemic chemotherapy to the same extent as other subtypes^5^, and not all ER-positive breast cancers respond optimally to endocrine therapy. Identifying patients with newly diagnosed cancer who are at a high risk of relapse despite standard chemo- and endocrine therapy could help select patients who require more effective treatments or provide a basis for recommending participation in clinical trials^6^.

The diversity in transcriptional programs accounts for much of the biological heterogeneity of breast cancer^7^. The luminal epithelial-specific genes, including ER and proliferation genes, are the main gene clusters differentially expressed among intrinsic subtypes^3^. Likewise, multigene prognostic assays primarily rely on ER and proliferation-related gene expression^8–16^. A meta-analysis of gene expression profiles from large cohorts revealed that the capacity of prognostic signatures depends mainly on the detection of proliferation activity, and that ER expression status may contain only indirect information about prognosis^16^. The Warburg effect of aerobic glycolysis, a key metabolic hallmark of cancer, fuels cell growth and proliferation. Positron emission tomography/computed tomography (PET/CT) using ^18^F-fluorodeoxyglucose allows visualization of the increased glucose metabolism in malignant tumors. In ER-positive, HER2-negative breast cancers, the maximum standardized uptake value (SUV) of ^18^F-fluorodeoxyglucose is associated with poor prognostic factors, including progesterone receptor status^17^, histologic grade, Ki-67 expression^18^, and 21-gene recurrence score^17^. ^18^F-fluorodeoxyglucose PET/CT provides prognostic information in a non-invasive manner and with a low overall cost when previously performed for staging. However, no previous studies have determined the ability of ^18^F-fluorodeoxyglucose PET/CT to predict distant relapse-free survival (DRFS) and overall survival (OS)^19–21^.

The main objective of this study was to examine the association of tumor ^18^F-fluorodeoxyglucose metabolism with DRFS in patients with ER-positive, HER2-negative breast cancer treated with anthracycline-based neoadjuvant chemotherapy (NCT) followed by adjuvant endocrine therapy. Administration of chemotherapy before surgery has an advantage over adjuvant chemotherapy in that it allows the differentiation of predictive markers of the response to chemotherapy from prognostic factors. Therefore, we also examined whether the maximum SUV was associated with pathological complete response (pCR) to NCT.

## Methods

### Study design, setting, and patients

This retrospective cohort study analyzed women with breast cancer treated with NCT, followed by surgery and adjuvant endocrine therapy between November 2007 and December 2014 at Asan Medical Center (Asan cohort), located in Seoul, Republic of Korea. Follow-up ended on December 31, 2019. Risk factors were examined in relation to outcomes that had already occurred at the start of the study. The institutional review board of Asan Medical Center approved the study protocol and waived the requirement for informed consent (2019-1171). The study was performed following the Declaration of Helsinki and institutional guidelines.

The study analyzed a consecutive series of women with ER-positive, HER2-negative breast cancer who received anthracycline-based NCT. ER status was considered positive if Allred scores were 3 or higher. Immunostaining for HER2 was considered positive in the case of strong (3+) membranous staining in at least 10% of tumor cells. In situ hybridization methods determined HER2 positivity of tumors with 2+ immunostaining. Patients had American Joint Committee on Cancer clinical stage II or III tumors (tumor stage ≥ T2) by American Joint Committee on Cancer staging with a histological type of invasive ductal carcinoma. Patients underwent ^18^F-fluorodeoxyglucose PET/CT and received at least one cycle of NCT. Patients were excluded if they had a prior history of cancer or bilateral breast cancer. The number of patients enrolled during the study period determined the sample size of the Asan cohort.

### ^18^F-fluorodeoxyglucose PET/CT

^18^F-fluorodeoxyglucose PET/CT imaging was performed from the skull base to the upper thigh at 50–70 min after intravenous administration of 5.2–7.4 MBq/kg (0.14–0.2 mCi/kg) of ^18^F-fluorodeoxyglucose^22^. The median blood glucose level before ^18^F-fluorodeoxyglucose injection was 102 mg/dl (IQR 94–109). Two board-certified nuclear medicine physicians who were blinded to patient outcomes drew a volume of interest on the primary breast cancer or metastatic lymph nodes and assessed the maximum SUV of ^18^F-fluorodeoxyglucose uptake. The maximum standardized uptake values were harmonized across various PET/CT scanners (Biograph Sensation 16 and Biograph TruePoint 40, Siemens Healthineers, Knoxville, TN, USA; Discovery STE 8, Discovery PET/CT 690, and Discovery PET/CT 710, GE Healthcare, Milwaukee, WI, USA) without partial volume correction^23^. The recovery coefficient profiles of variable-sized hot cylinders of the American College of Radiology-approved PET phantoms (i.e., Esser phantom) were matched^24,25^. Annual ^18^F water cylinder phantom-based cross-calibration between PET and dose calibrator assured the uniform standardized uptake value of 1.0 between PET scanners^25^.

### Variables

The primary outcome measure of this study was DRFS. The secondary outcome was OS. DRFS was defined as the interval from the date of NCT to the diagnosis of distant metastasis or death from breast cancer, non-breast cancer, or unknown causes^26,27^. OS was measured until the date of death from any cause. Pathologic complete response (pCR) was defined as the absence of residual invasive cancer on hematoxylin and eosin-stained samples of the complete resected breast specimen and all sampled regional lymph nodes^26,28–30^. Factors considered potential predictors of DRFS and OS that were prespecified in the study protocol were age, tumor stage, clinical lymph node stage, histologic grade, ER Allred score, progesterone receptor status, and Ki-67 expression^31–33^, as previously described^26,28,29,34–36^. All variables prior to NCT were retrieved from the electronic medical records of Asan Medical Center. Predictors that had continuous values or belonged to three or more categories were dichotomized based on commonly used cut-off values that are relevant for prognosis as follows: age (20–50 vs. > 50)^31–33^, tumor stage (T1–2 vs. T3–4)^31–33^, clinical N stage (N0 vs. N1–3)^31–33^, histologic grade (1 or 2 vs. 3)^37,38^, ER score (3–6 vs. 7–8)^31,39^, and Ki-67 expression (< 20% vs. ≥ 20%)^31,40,41^. The maximum SUV was analyzed as a continuous variable or grouped according to the median or tertile.

### Statistical analysis

Data were expressed as the median and interquartile range (IQR) for continuous variables or numbers (%) for categorical variables. A two-sided *P* value of < 0.05 was considered significant. Categorical variables were compared using the χ^2^ test. For continuous variables, the Mann–Whitney U test was used. Associations were analyzed using linear regression analysis. Survival curves were estimated using the Kaplan–Meier method and compared with the log-rank test. Logistic regression analysis was used to determine the predictors of pCR, and Cox proportional hazard regression analysis was applied to identify independent predictors of DRFS and OS based on a predetermined statistical plan. Patients were censored to the date of the last disease assessment. For the multivariable analysis, a model was constructed including clinical and pathological predictors that were associated with the outcome at *P* ≤ 0.10. Post hoc analysis was performed to assess whether the overall association was consistent across all subgroups studied. For logistic and Cox regression, linearity, potential influential observations, and multicollinearity were assessed. The proportional hazard assumption was tested with Schoenfeld’s residual test and Schoenfeld’s partial residual plots. A restricted cubic spline plot was used for detecting nonlinearity and deviance residual for examining influential observations. Whether an overall association was consistent across all subgroups of patients categorized according to variables for survival analysis was examined. Estimates were presented with a 95% confidence interval (CI).

Missing clinical and pathological data were imputed using the multiple imputation method (m = 10). Ten imputed data sets were generated, and estimates from the multiple imputed data sets were combined according to Rubin’s rule^42^. The robustness of the results was demonstrated by performing a complete case analysis as a sensitivity analysis of the imputation model. Statistical tests were performed using R statistical software, version 3.6.1 (The R Foundation for Statistical Computing) and IBM SPSS Statistics for Windows, version 21 (IBM Corporation).

## Results

### Patient characteristics

**Figure 1:**
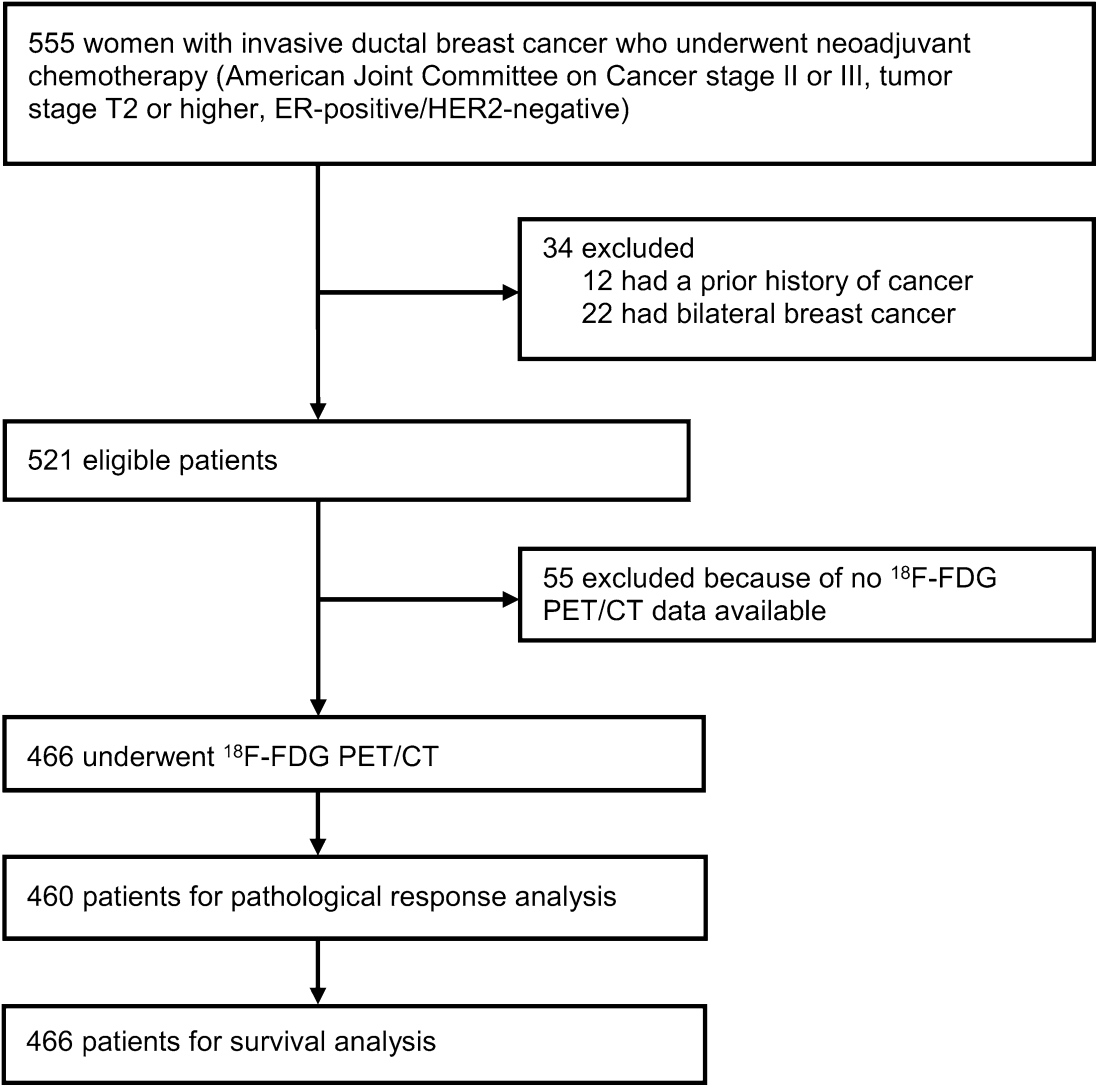
Flow diagram. Pathological complete response and survival were investigated in the Asan Medical Center cohort of ER-positive, HER2-negative breast cancer patients.

**Figure 2:**
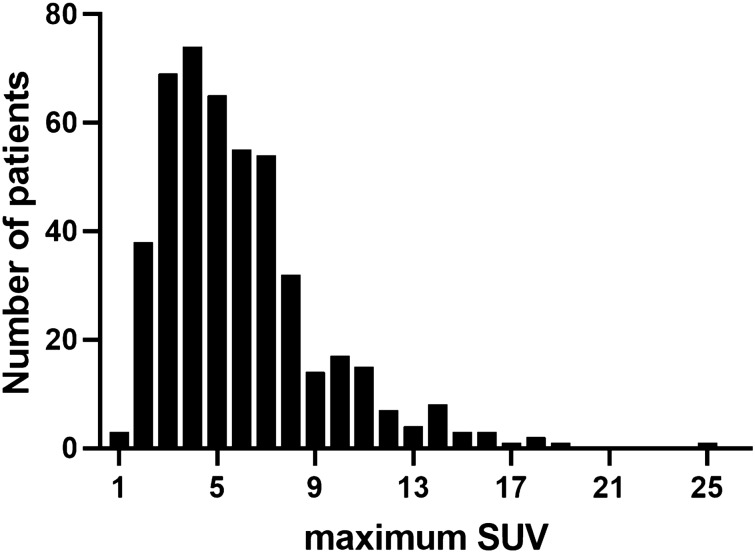
Distribution of maximum SUV of ^18^F-fluorodeoxyglucose PET/CT in patients with breast cancer.

The study cohort comprised 466 women with breast cancer (Fig. 1). Patients’ demographics were typical of the neoadjuvant setting, including patients with regional lymph node disease or T3–4 tumors (Table 1). The median time between ^18^F-fluorodeoxyglucose PET/CT and the first date of NCT was 8 days (IQR 6–10). The median age of enrolled patients was 45 years (IQR 39–51). Patients received anthracycline (anthracycline and cyclophosphamide, n = 125, 26.8%) or taxane-based NCT (anthracycline, cyclophosphamide and taxane, n = 341, 73.2%) followed by breast-conserving surgery (n = 230, 49.4%) or mastectomy (n = 230, 49.4%). The clinical and pathological characteristics of enrolled patients are shown in Table 1. All patients except five who were lost to follow-up (n = 4) or had ER-negative cancer after surgery (n = 1) received adjuvant tamoxifen (n = 366, 78.5%) or aromatase inhibitor therapy (n = 95, 20.4%). The clinical and pathological characteristics of patients who did not undergo ^18^F-fluorodeoxyglucose PET/CT before NCT were not significantly different from those of patients who underwent ^18^F-fluorodeoxyglucose PET/CT (Supplementary Table S1). The maximum SUV ranged from 1.36 to 25.06, with a median value of 5.14 (Fig. 2), and was associated with clinical characteristics (Table 2).

**Table 1:** Clinical and pathological characteristics (n = 466).

Characteristics	*n* (%)
**Age, years**	
20–50	341 (73.2%)
> 50	125 (26.8%)
**Tumor stage**	
T2	322 (69.1%)
T3–4	144 (30.9%)
**Clinical N stage**	
N0	155 (33.3%)
N1–3	311 (66.7%)
**Histologic grade**	
G1–2	401 (86.1%)
G3	63 (13.5%)
Unknown	2 (0.4%)
**ER score (Allred)**	
3–6	62 (13.3%)
7–8	404 (86.7%)
**Progesterone receptor status**	
Negative	82 (17.6%)
Positive	384 (82.4%)
**Ki-67**	
< 20%	133 (28.6%)
≥ 20%	290 (62.2%)
Unknown	43 (9.2%)
**NCT response**	
pCR	22 (4.7%)
No pCR	438 (94.0%)
Unknown	6 (1.3%)^a^
**DRFS data**^**b**^	
Censored	382 (82.0%)
Yes	84 (18.0%)
**OS data**^**c**^	
Censored	414 (88.8%)
Yes	52 (11.2%)

^a^Number of patients who did not undergo surgical treatment because of loss to follow-up (n = 4), refusal of surgery (n = 1), and distant relapse (n = 1).

^b^Number of patients with distant metastasis or death.

^c^Number of patients with death.

**Table 2:** Association between ^18^F-fluorodeoxyglucose metabolism of breast cancer and other characteristics.

Characteristics	Maximum SUV^a^	*P* value
**Age, years**		
20–50 vs. > 50	0.09 (− 0.59 to 0.77)	0.80
**Tumor stage**		
T1–2 vs. T3–4	0.36 (− 0.29 to 1.01)	0.28
**Clinical N stage**		
N0 vs. N1–3	1.03 (0.40 to 1.66)	0.001
**Histologic grade**		
G1–2 vs. G3	3.19 (2.35 to 4.02)	< 0.001
**ER score (Allred)**		
3–6 vs. 7–8	− 2.87 (− 3.71 to − 2.02)	< 0.001
**Progesterone receptor status**		
Negative vs. positive	− 1.55 (− 2.32 to − 0.77)	< 0.001
**Ki-67 expression**		
< 20% vs. ≥ 20%	1.63 (0.99 to 2.27)	< 0.001

^a^Data represent the regression coefficients (95% confidence interval).

### Response to NCT

Of 460 patients who underwent surgery, 22 (4.8%) achieved a pCR. Univariable analysis showed that only progesterone receptor status (odds ratio 0.19; 95% CI 0.08–0.45, *P* < 0.001) was significantly associated with pCR (Supplementary Table S2). Multivariable analysis, including histologic grade and progesterone receptor, also showed that positivity for progesterone receptor was independently associated with pCR (odds ratio 0.21; 95% CI 0.08–0.51; *P* = 0.001).

### Prognostic value

**Figure 3:**
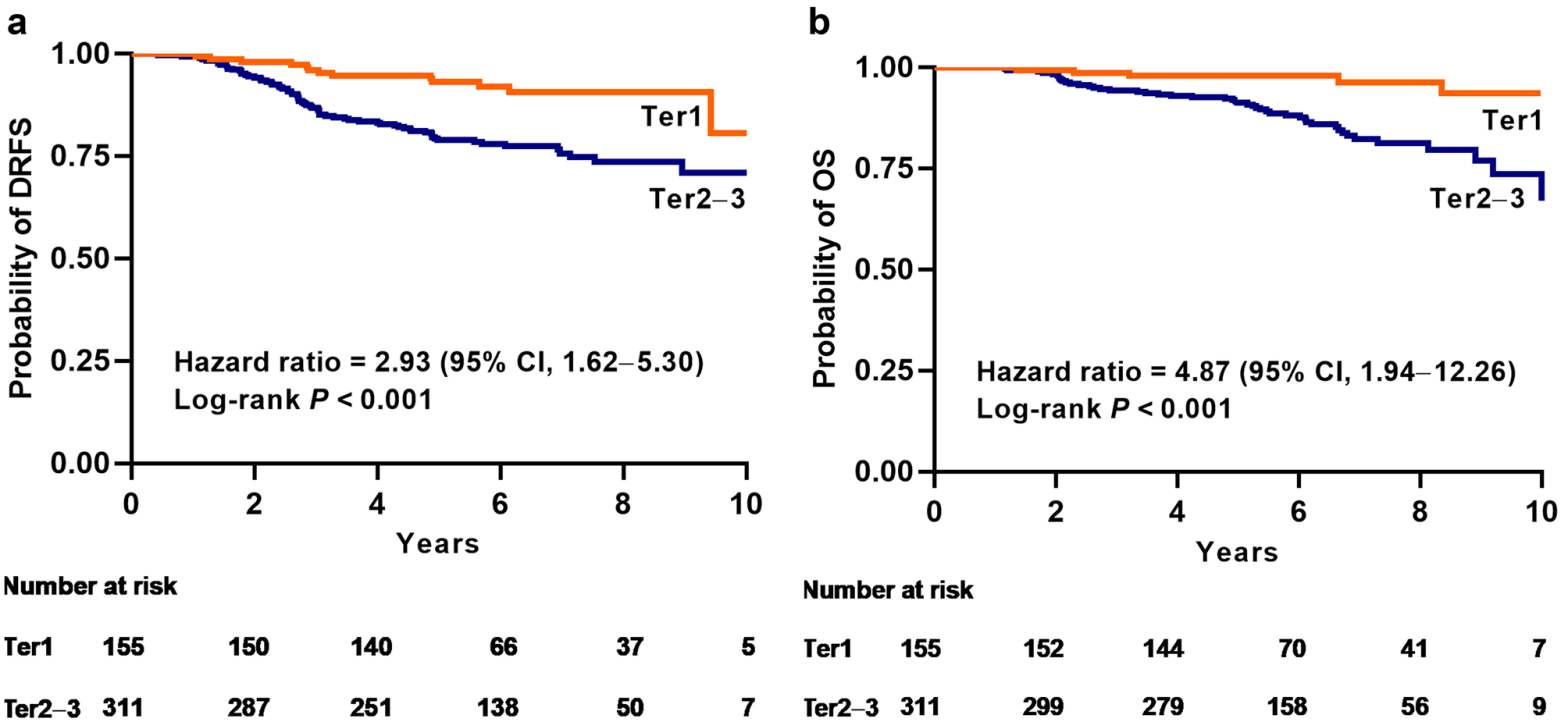
Kaplan–Meier curves of DRFS and OS by tertiles of the maximum standardized uptake value of ^18^F-fluorodeoxyglucose. The middle and high tertile categories were combined into one high-risk group because their outcome was significantly different from the low tertile. (**a**) The middle and high tertiles of SUV were prognostic for DRFS (hazard ratio 2.93, 95% confidence interval [CI] 1.62–5.30; *P* < 0.001). The 8-year DRFS rates were 90.7% (95% CI 85.5–96.1%) for those in the low tertile of maximum SUV vs. 73.7% (95% CI 68.0–79.8%) for those in the middle and high tertiles of maximum SUV. (**b**) The middle and high tertiles of SUV were prognostic for OS (hazard ratio 4.87, 95% CI 1.94–12.26; *P* < 0.001). The 8-year OS rates were 96.4% (95% CI 92.6–100%) for those in the low tertile of maximum SUV vs. 81.3% (95% CI 76.0–87.0%) for those in the middle and high tertiles of maximum SUV. *DRFS* distant relapse-free survival, *OS* overall survival, *SUV* standardized uptake value, *Ter1* low tertile, *Ter2–3* middle and high tertiles.

The median follow-up period for patients without distant metastasis or death was 6.2 (IQR 5.3–7.6) years. Distant metastasis or death did not occur in the 22 patients who achieved a pCR. Patients with pCR had significantly longer DRFS (*P* = 0.04, Supplementary Fig. S1) than those who did not achieve pCR, but there was no significant difference in OS (*P* = 0.15). Age, tumor stage, clinical N stage, Ki-67 expression, and maximum SUV (continuous and categorical) were associated with DRFS and OS (Table 3). Progesterone receptor status was also associated with OS. Multivariable analysis showed that maximum SUV was independently associated with DRFS and OS when analyzed as a categorical estimate by grouping the patients according to the median or tertile value (Table 4). The tumor stage and clinical N stage were also independent prognostic factors for DRFS and OS. Ki-67 expression was not independently associated with DRFS and OS regardless of whether we analyzed it as a continuous variable or several ordered categories. Kaplan–Meier estimates for patients who were free of distant metastasis or death were significantly different according to the maximum SUV (*P* < 0.001, Fig. 3). Post-hoc analysis of DRFS and OS showed that the overall association between maximum SUV and survival was consistent across most clinical and pathological subgroups large enough to provide sufficient outcome data (Supplementary Figs. S2, S3). When patients were stratified into subgroups according to independent prognostic factors, the increase in absolute risk of distant metastasis and death associated with high maximum SUV applied predominantly to patients with clinically node-positive disease (Supplementary Figs. S4, S5).

**Table 3:** Univariable Cox proportional hazard analyses for survival (n = 466).

Characteristic	DRFS		OS	
	HR (95% CI)	*P* value	HR (95% CI)	*P* value
Age, years: 20–50 vs. > 50	1.64 (1.05–2.57)	0.03	2.12 (1.22–3.70)	0.008
Tumor stage: T2 vs. T3–4	1.74 (1.13–2.68)	0.01	1.94 (1.12–3.36)	0.02
Clinical N stage: N0 vs. N1–3	4.03 (2.08–7.81)	< 0.001	8.56 (2.67–27.47)	< 0.001
Histologic grade: G1–2 vs. G3	1.07 (0.58–1.98)	0.82	1.73 (0.89–3.38)	0.11
ER score (Allred): 3–6 vs. 7–8	0.90 (0.49–1.65)	0.73	0.75 (0.36–1.53)	0.42
PR status: negative vs. positive	0.64 (0.39–1.06)	0.09	0.50 (0.28–0.91)	0.02
Ki-67 expression: < 20% vs. ≥ 20%	2.48 (1.26–4.88)	0.009	4.63 (1.53–13.98)	0.007
Maximum SUV: continuous	1.08 (1.02–1.14)	0.01	1.13 (1.06–1.21)	< 0.001
Maximum SUV: < 5.14 vs. ≥ 5.14^a^	2.21 (1.40–3.48)	0.001	3.70 (1.94–7.07)	< 0.001
Maximum SUV: Ter1 vs. Ter2	2.42 (1.26–4.63)	0.008	2.78 (1.00–7.71)	0.05
Maximum SUV: Ter1 vs. Ter3	3.48 (1.87–6.50)	< 0.001	7.19 (2.80–18.45)	< 0.001

*HR* hazard ratio, *PR* progesterone receptor, *Ter1* low tertile of SUV (1.36–4.14), *Ter2* middle tertile of SUV (4.14–6.62), *Ter3* high tertile of SUV (6.70–25.06).

^a^Maximum SUV was dichotomized by the median value.

**Table 4:** Multivariable Cox proportional hazard analyses for DRFS and OS (n = 466).

Characteristic		DRFS		OS	
		Hazard ratio (95% CI)	*P* value	Hazard ratio (95% CI)	*P* value
Age, years: 20–50 vs. > 50		1.50 (0.95–2.37)	0.09	2.01 (1.13–3.58)	0.02
Tumor stage: T2 vs. T3–4		1.66 (1.06–2.58)	0.03	1.81 (1.02–3.20)	0.04
Clinical N stage: N0 vs. N1–3		3.01 (1.54–5.89)	0.001	6.21 (1.91–20.18)	0.002
Histologic grade: G1–2 vs. G3		0.76 (0.41–1.43)	0.40	1.06 (0.53–2.11)	0.87
PR status: negative vs. positive		0.75 (0.45–1.25)	0.27	0.58 (0.31–1.07)	0.08
Ki-67 expression: < 20% vs. ≥ 20%		1.75 (0.88–3.48)	0.11	3.11 (0.97–9.99)	0.06
Maximum SUV^a,b^	Ter1 vs. Ter2	2.26 (1.17–4.39)	0.02	3.01 (1.05–8.58)	0.04
	Ter1 vs. Ter3	2.93 (1.55–5.54)	0.001	6.62 (2.49–17.58)	< 0.001

*PR* progesterone receptor, *Ter1* low tertile of SUV (1.36–4.14), *Ter2* middle tertile of SUV (4.14–6.62), *Ter3* high tertile of SUV (6.70–25.06).

^a^Hazard ratio (95% CI) of maximum SUV as a continuous measurement: 1.01 (0.99 to 1.12, *P* = 0.09) for DRFS, and 1.12 (1.04–1.20, *P* = 0.003) for OS.

^b^Hazard ratio (95% CI) of maximum SUV as a categorical estimate according to the median value (< 5.14 vs. ≥ 5.14): 2.00 (1.25–3.18, *P* = 0.004) for DRFS and 3.58 (1.82–7.05, *P* < 0.001) for OS.

## Discussion

The present study demonstrated that high tumor ^18^F-fluorodeoxyglucose metabolism was associated with reduced DRFS and OS after adjusting for standard prognostic factors in patients with ER-positive, HER2-negative breast cancer treated with anthracycline-based NCT, followed by adjuvant endocrine therapy. To the best of our knowledge, this study is the first to demonstrate the value of tumor ^18^F-fluorodeoxyglucose metabolism for the long-term prognosis of DRFS and OS in patients with ER-positive, HER2-negative breast cancer. ^18^F-fluorodeoxyglucose PET/CT may be helpful in selecting patients who need close monitoring or novel investigational treatment. In this study, maximum SUV was evaluated as a variable to assess tumor ^18^F-fluorodeoxyglucose metabolism because it is the most studied and most readily available routinely to assess tumor biology^43^. The harmonized SUV values from different PET scanners were obtained in patients with T2 or higher tumors. The strength of this study is that it studied a large and homogenous population with ER-positive, HER2-negative breast cancer patients.

Our results showed that negative progesterone receptor status was associated with a higher rate of pCR. However, the association with pCR appeared to be the opposite of its association with poor survival. Negative progesterone receptor status was associated with worse OS despite the higher chemotherapy sensitivity in these patients. The number of patients who achieved a pCR was small, and the rate of poor prognosis was mainly determined by those who did not achieve pCR. Previous studies investigating ER-positive breast cancer obtained the same results for multigene prognostic signatures^26,44–46^. This paradox is attributable to the relationship between the biological information provided by the predictor and the frequency and prognosis of false-positive predictions^26^. Less differentiated tumors are more likely to respond to chemotherapy and have a poor prognosis if they do not respond. These observations highlight that in ER-positive, HER2-negative breast cancer, survival is influenced not only by chemotherapy response but also by baseline biologic features and sensitivity to endocrine therapy^45^. Therefore, NCT that modestly increases the pCR rate is unlikely to improve prognosis^47^. In this study, the increased sensitivity to chemotherapy did not fully compensate for the poor baseline prognosis and low sensitivity to endocrine therapy. Meanwhile, ^18^F-fluorodeoxyglucose metabolism failed to show a significant association with pCR. Therefore, although ^18^F-fluorodeoxyglucose PET/CT was performed in the NCT setting, ^18^F-fluorodeoxyglucose metabolism in this study may reflect baseline prognostic features.

An important question is whether the results obtained in the neoadjuvant setting can be applied to the adjuvant setting. One limitation in this regard is that the prognostic information provided by ^18^F-fluorodeoxyglucose metabolism was obtained from a large number of patients with advanced clinical stages. Gene expression studies indicate that primary tumor samples and metastatic lymph node samples from the same individual are usually more similar to each other than to other samples, suggesting that the molecular program of primary breast cancer is retained in nodal metastases^7^. Furthermore, multigene assays have a prognostic value independent from the presence or absence of lymph node involvement^48–51^. These results suggest that ^18^F-fluorodeoxyglucose metabolism, a marker of cell growth and proliferation, can be used to identify a high-risk population in the adjuvant setting when prognostic gene expression data are not available.

The present study had several limitations. First, the outcome data collected was based on events that had already occurred at the start of the study. However, this study included all known predictors and potential confounders. The statistical methods were predetermined according to the primary objectives in the study protocol. Only a small number of patients did not undergo PET/CT, and there was no significant difference in characteristics between patients who did or did not undergo staging ^18^F-fluorodeoxyglucose PET/CT. Therefore, the possibility of information or selection bias was minimal. Second, we did not establish the cut-off value of the maximum SUV to define good and poor prognosis for patients. Prognostic characteristics of the cut-off value should be demonstrated on the independent data sets. The present results indicate that the harmonized maximum SUV of 4.1, which was the low tertile value, could be selected for a validation study based on the significance of the split in the survival curve.

In conclusion, high tumor ^18^F-fluorodeoxyglucose metabolism was associated with reduced DRFS and OS after adjusting for standard prognostic factors. ^18^F-fluorodeoxyglucose PET/CT may help classify ER-positive, HER2-negative patients into groups that would benefit from different therapeutic options.

## Supplementary Information

Supplementary Information.

## Data Availability

The datasets generated during and/or analyzed during the current study are available from the corresponding author on reasonable request.
