# Supplementary material for: Association between tumor 18F-fluorodeoxyglucose metabolism and survival in women with estrogen receptor-positive, HER2-negative breast cancer
Source: Sci Rep. 2022 May 12;12:7858. doi: 10.1038/s41598-022-11603-z (PMC9098458; doi:10.1038/s41598-022-11603-z)
Supplement: Supplementary file 1 — Supplementary Information. [file 41598_2022_11603_MOESM1_ESM.pdf]

SUPPLEMENTARY INFORMATION

Association between tumor ^{18}F-fluorodeoxyglucose metabolism and survival in women with estrogen receptor-positive, HER2-negative breast cancer

Sun Young Chae,[1] Seol Hoon Park,[2] Hyo Sang Lee,[3] Jin-Hee Ahn,[4] Sung-Bae Kim,[4] Kyung Hae Jung,[4] Jeong Eun Kim,[4] Sei Hyun Ahn,[5] Byung Ho Son,[5] Jong Won Lee,[5] Beom Seok Ko,[5] Hee Jeong Kim,[5] Gyungyub Gong,[6] Jungsu S. Oh,[7] Seo Young Park,[8#] Dae Hyuk Moon[7]

[1]Department of Nuclear Medicine, Uijeongbu Eulji Medical Center, Eulji University School of Medicine

[2]Department of Nuclear Medicine, Ulsan University Hospital, University of Ulsan College of Medicine, Ulsan, Republic of Korea

[3]Department of Nuclear Medicine, GangNeung Asan Hospital, University of Ulsan College of Medicine, Gangneung, Republic of Korea

[4]Department of Oncology, Asan Medical Center, University of Ulsan College of Medicine, Seoul, Republic of Korea

[5]Department of Surgery, Asan Medical Center, University of Ulsan College of Medicine, Seoul, Republic of Korea

[6]Department of Pathology, Asan Medical Center, University of Ulsan College of Medicine, Seoul, Republic of Korea

[7]Department of Nuclear Medicine, Asan Medical Center, University of Ulsan College of Medicine, Seoul, Republic of Korea

[8#]Department of Clinical Epidemiology and Biostatistics, Asan Medical Center, University of Ulsan College of Medicine, Seoul, Republic of Korea

[#]Current address: Department of Statistics and Data Science, Korea National Open University, Seoul, Republic of Korea

Supplementary Tables

Supplementary Table S1. Comparison of clinical and pathological characteristics between patients who did and did not undergo ^{18}F-fluorodeoxyglucose positron emission tomography/computed tomography before neoadjuvant chemotherapy

**Characteristics**	**^{18}F-FDG PET/CT (n = 466)**	**No ^{18}F-FDG PET/CT (n = 55)**	***P* value**
Age, years			
20–50	341 (73.2%)	45 (81.8%)	0.22
>50	125 (26.8%)	10 (18.2%)	
Tumor stage			
T1–2	322 (69.1%)	38 (69.1%)	1.00
T3–4	144 (30.9%)	17 (30.9%)	
Clinical N stage			
N0	155 (33.3%)	17 (30.9%)	0.84
N1–3	311 (66.7%)	38 (69.1%)	
Histologic grade			0.72
G1–2	401 (86.4%)	46 (83.6%)	
G3	63 (13.6%)	8 (14.6%)	
Unknown	2 (0%)	1 (1.8%)	
ER score (Allred)			1.00
3–6	62 (13.3%)	7 (12.7%)	
7–8	404 (86.7%)	48 (87.3%)	
Progesterone receptor status			0.08
Negative	82 (17.6%)	4 (7.3%)	
Positive	384 (82.4%)	51 (92.7%)	
Ki-67 expression			
<20%	133 (28.5%)	17 (30.9%)	0.31
≥20%	290 (62.2%)	24 (43.6%)	
Unknown	43 (9.2%)	14 (25.5%)	

^{18}F-FDG = ^{18}F-fluorodeoxygluose.

Supplementary Table S2. Univariable analysis for pCR (n = 460)

Characteristic	Odds ratio (95% CI)	*P* value
Age, years: 20–50 vs. >50	0.81 (0.26–2.09)	0.68
Tumor stage: T2 vs. T3–4	1.06 (0.40–2.57)	0.90
Clinical N stage: N0 vs. N1–3	1.35 (0.54–3.82)	0.54
Histologic grade: G1–2 vs. G3	2.58 (0.97–6.89)	0.06
ER score (Allred): 3–6 vs. 7–8	0.50 (0.19–1.56)	0.19
Progesterone receptor status: negative vs. positive	0.19 (0.08–0.45)	< 0.001
Ki-67 expression: <20% vs. ≥20%	1.73 (0.56–5.38)	0.34
Maximum SUV, continuous	1.01 (0.87–1.13)	0.93
[a]Maximum SUV, <5.14 vs. ≥ 5.14	0.84 (0.35–1.99)	0.69
Maximum SUV: Ter1 vs. Ter2	0.66 (0.22–1.87)	0.44
Ter1 vs. Ter3	0.77 (0.27–2.13)	0.62

[a]Maximum SUV was dichotomized by the median value. Ter1 = low tertile of SUV (1.36–4.14); Ter2 = middle tertile of SUV (4.14–6.62); Ter3 = high tertile of SUV (6.70–25.06).

Supplementary Figures

Supplementary Figure S1. Kaplan-Meier estimates of DRFS and OS of breast cancer patients according to pCR after NCT.

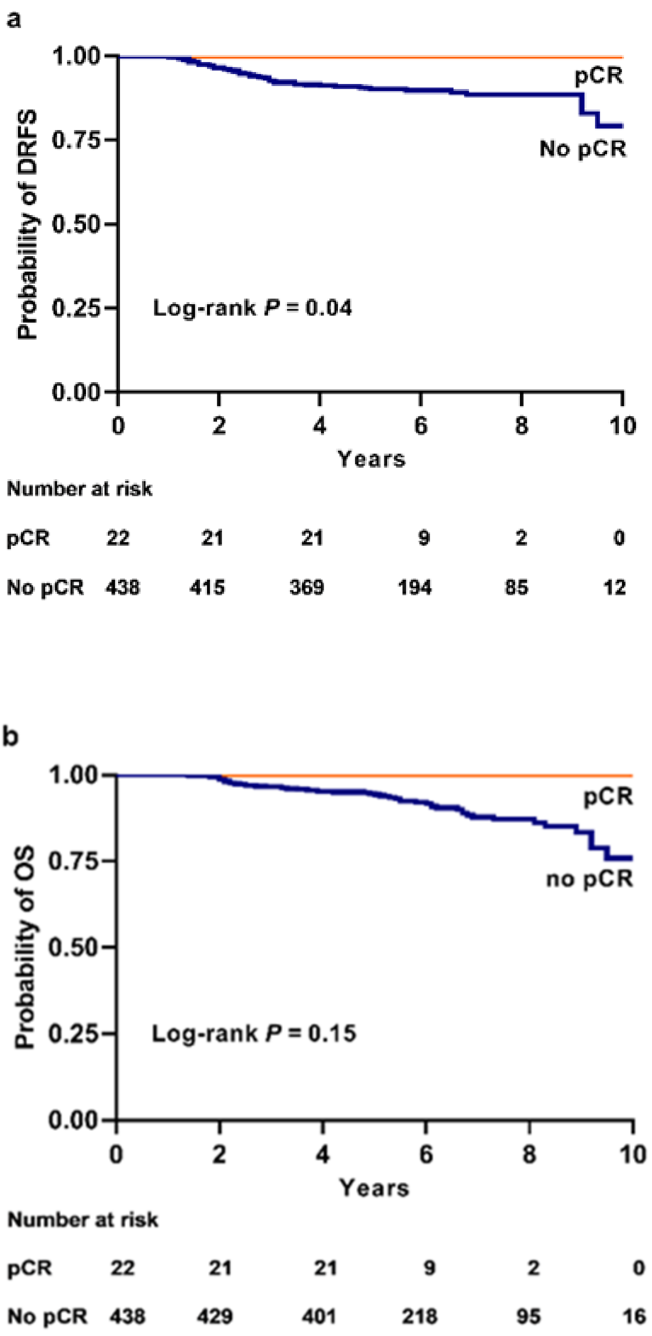

(a) Distant relapse occurred in none of 22 patients who achieved pCR. However, among 438 patients who did not achieve pCR, distant relapse occurred in 81 (18.5%) patients, and the difference between the groups was significant (*P* = .04). (b) No death

was observed among 22 patients who achieved pCR, whereas 49 (11.1%) patients who did not achieve pCR died during the follow-up period.

Supplementary Figure S2. Extended Cox proportional hazard analyses of maximum SUV for DRFS.

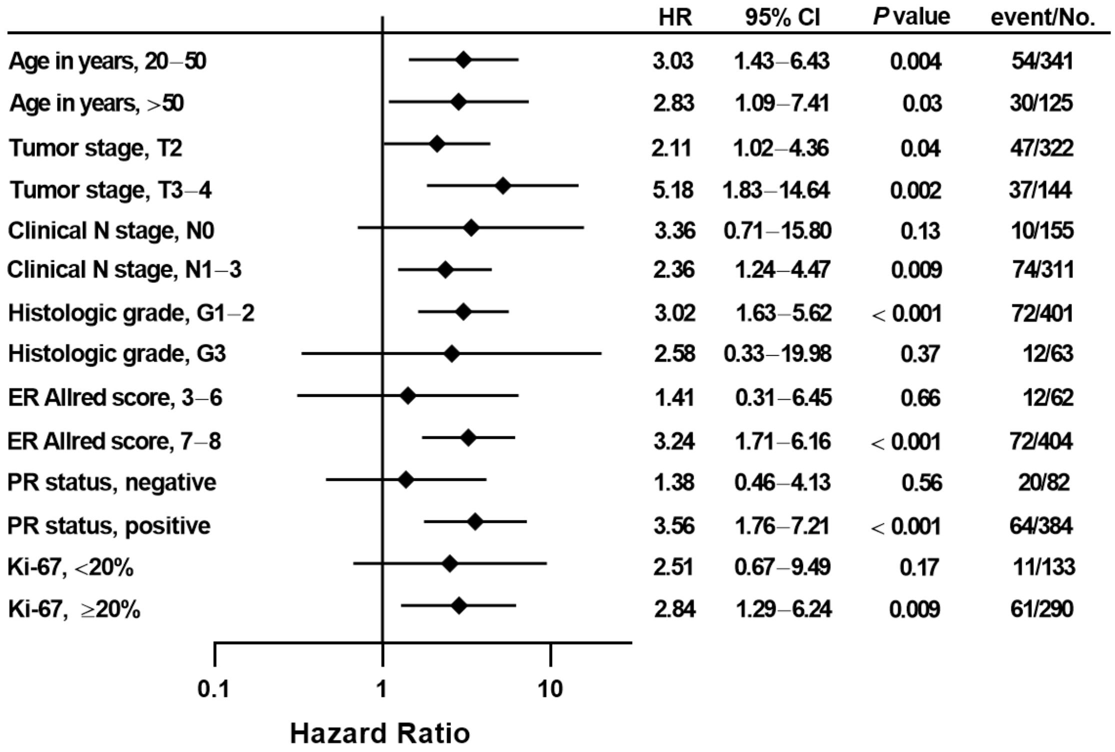

Subgroup analysis evaluating the association between the maximum standardized uptake value and distant relapse-free survival according to clinical and pathological characteristics. The maximum standardized uptake value was dichotomized based on the cut-off value of the low tertile (the low tertile vs. the middle and high tertiles).

Supplementary Figure S3. Extended Cox proportional hazard analyses of maximum SUV for OS

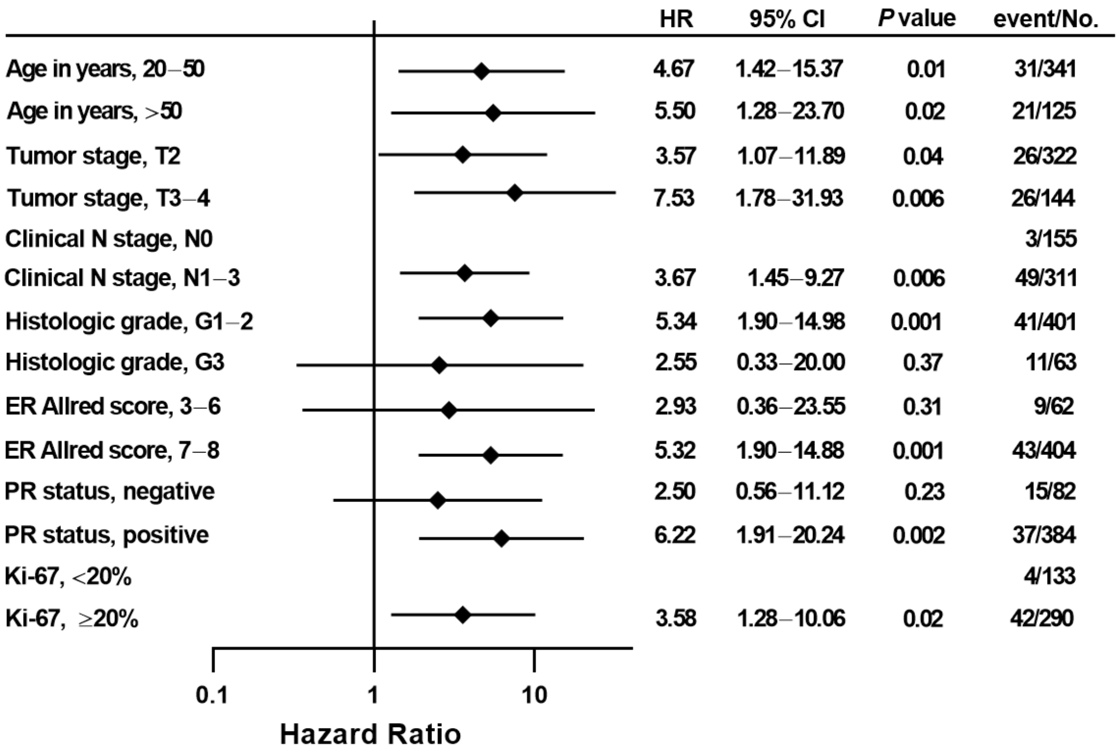

Subgroup analysis evaluating the association between the maximum standardized uptake value and overall survival according to clinical and pathological characteristics. The maximum standardized uptake value was dichotomized based on the cut-off value of the low tertile (the low tertile vs. the middle and high tertile).

Supplementary Figure S4. Kaplan-Meier curves of DRFS according to tertiles of the SUV of ^{18}F-fluorodeoxyglucose PET/CT.

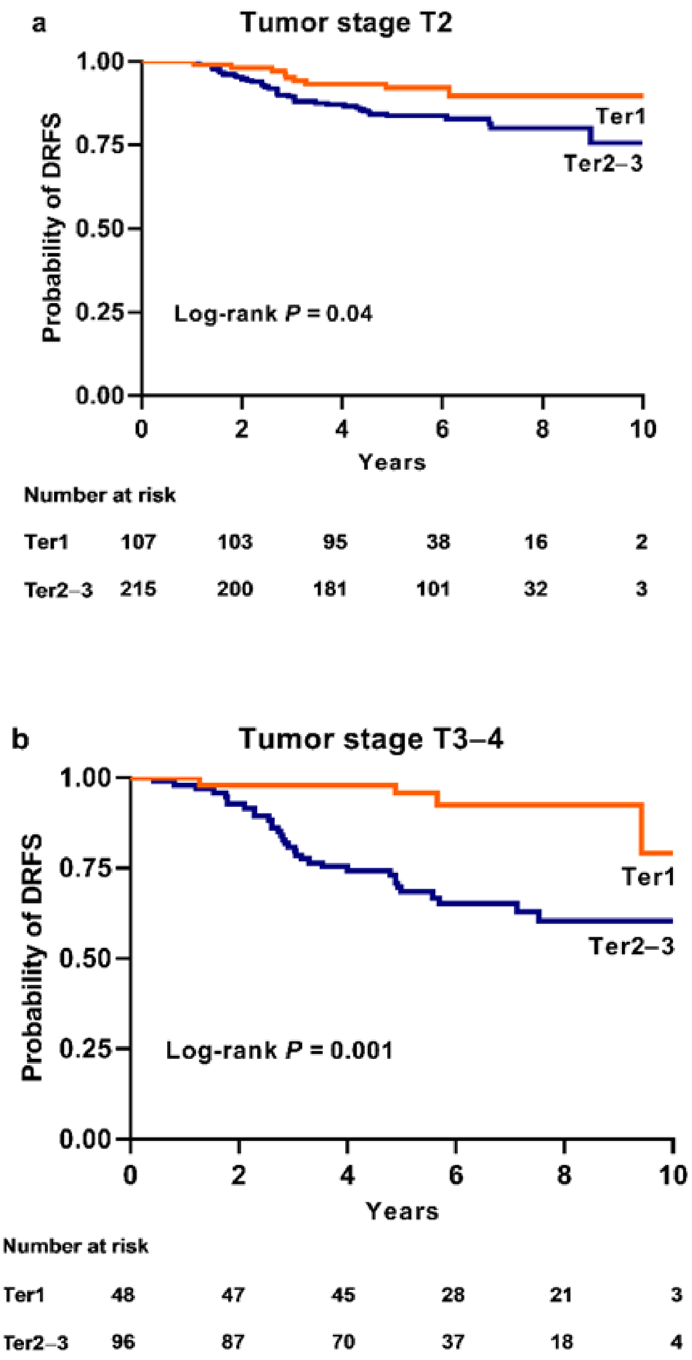

Supplementary Figure S4. Kaplan-Meier curves of DRFS according to tertiles of the maximum SUV of ^{18}F-fluorodeoxyglucose PET/CT (continued).

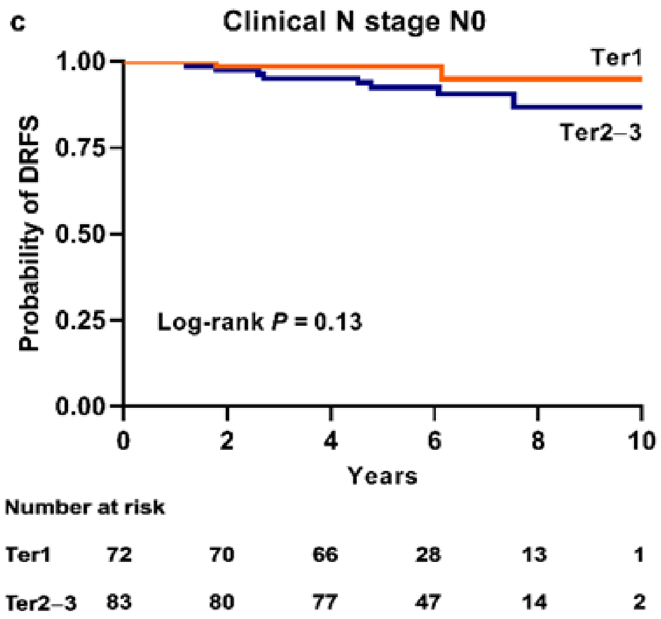

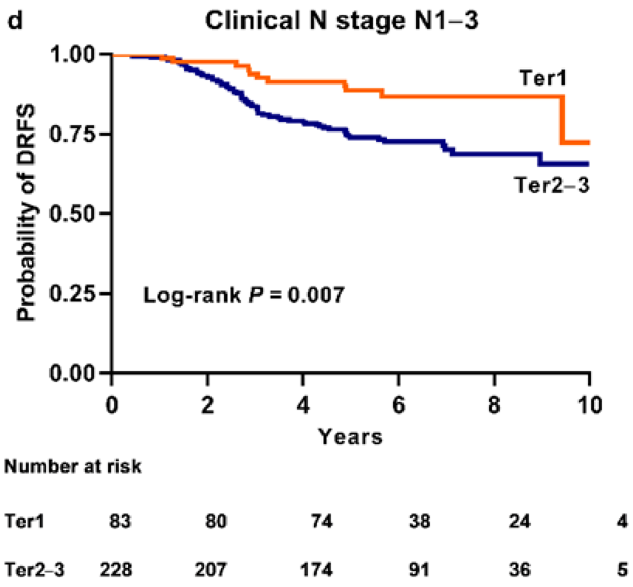

Subgroup analysis evaluating the association between the maximum SUV and DRFS according to tumor stage (a: tumor stage T2; b: tumor stage T3–4) and clinical N stage (c: node-negative disease, N0; d: node-positive disease, N1–3). The maximum SUV was dichotomized based on the cut-off value of the low tertile.Ter1 indicates a subgroup of patients in the low tertile of SUV (1.36–4.14); Ter2-3 represents patients with the middle and high tertiles of SUV (4.14–25.06). The 8-year DRFS rates (with 95% CI) of patients in the low tertile of the maximum SUV vs. those in the middle, or the high tertile of SUVmax were (a) 89.7% (83.0–96.9%) vs. 80.1% (74.0–86.6%), (b) 92.4% (84.3–100.0%) vs. 60.3% (49.9–72.8%), (c) 94.9% (87.7–100%) vs. 86.8% (77.6–97.1%), and (d) 86.8% (79.4–94.9%) vs. 68.8% (62.0–76.3%).

Supplementary Figure S5. Kaplan-Meier curves of OS according to tertiles of the maximum SUV of ^{18}F-fluorodeoxyglucose PET/CT.

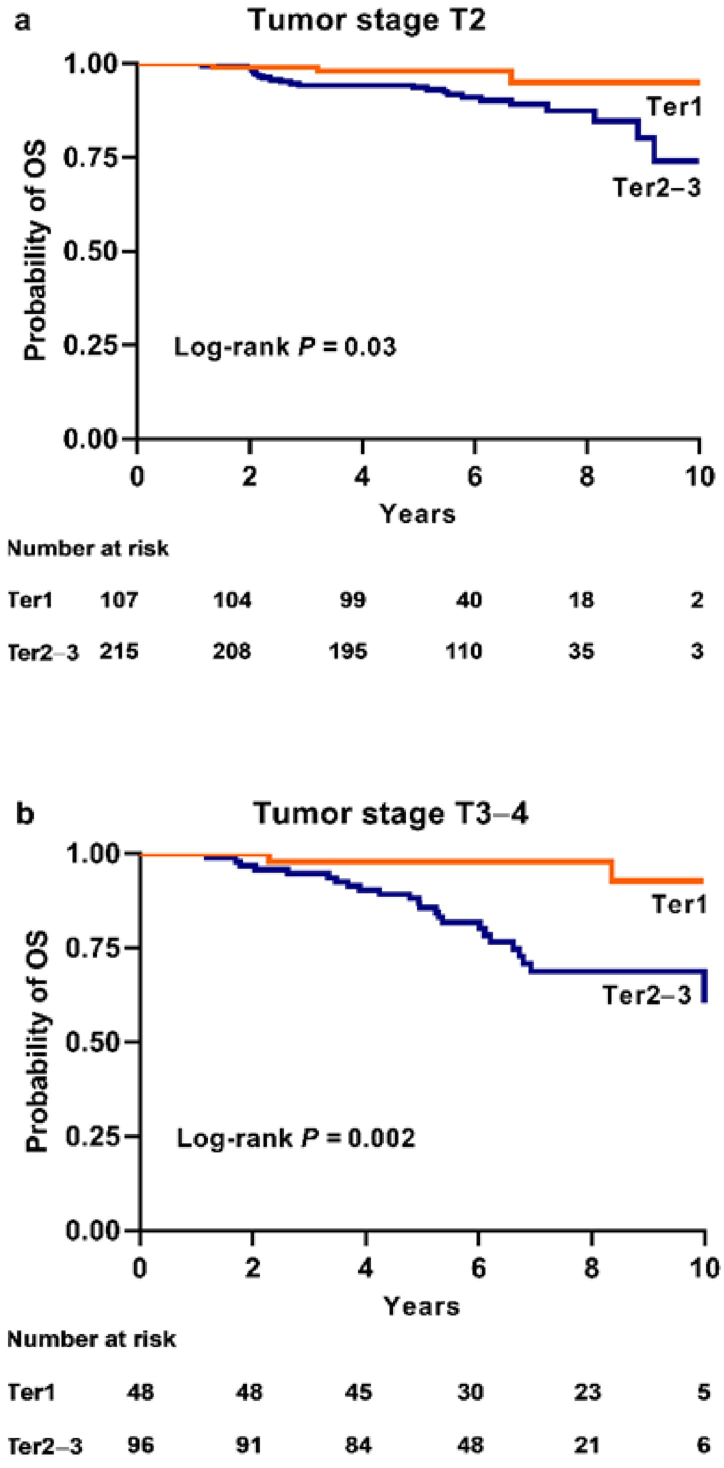

Supplementary Figure S5. Kaplan-Meier curves of OS according to tertiles of the maximum SUV of ^{18}F-fluorodeoxyglucose PET/CT (continued).

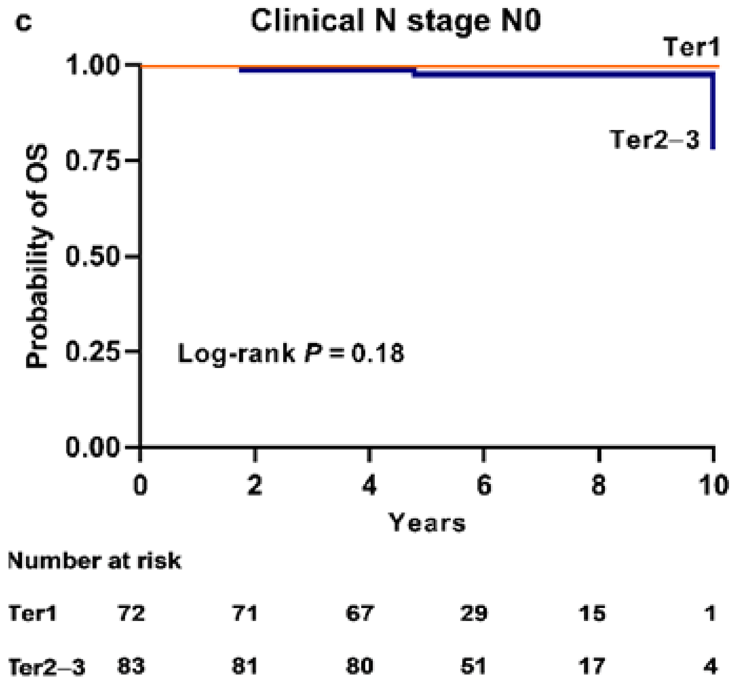

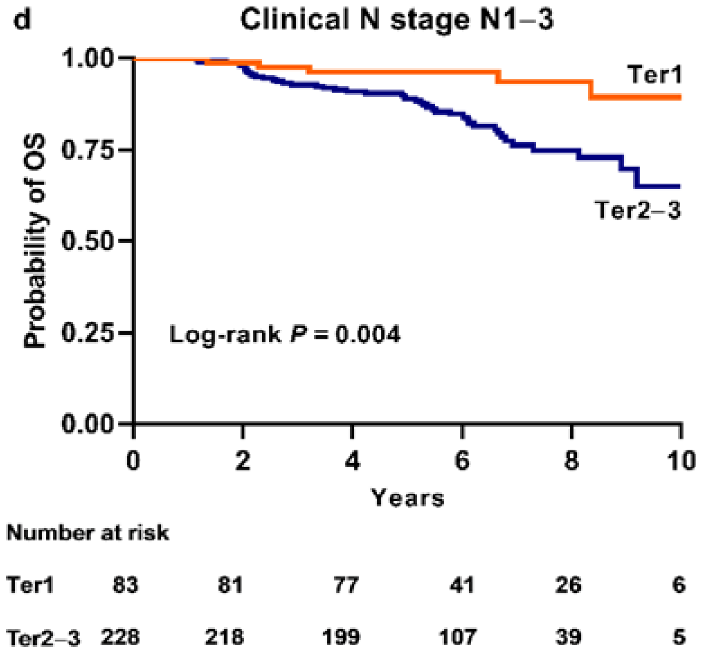

Subgroup analysis evaluating the association between the maximum SUV and OS according to tumor stage (a: tumor stage T2; b: tumor stage T3–4) and clinical N stage (c: node-negative disease, N0; d: node-positive disease, N1–3). The maximum SUV was dichotomized based on the cut-off value of the low tertile. Ter1 indicates a subgroup of patients in the low tertile of SUV (1.36–4.14); Ter2–3 represents patients in the middle and high tertiles of SUV (4.14–25.06). The 8-year OS rates (with 95% CI) of patients in the low tertile of the maximum SUV vs. those in the middle, or the high tertile of SUVmax were (a) 95.0% (88.8–100.0%) vs. 87.5% (81.9–93.4%), (b) 97.9% (94.0–100.0%) vs. 68.9% (58.6–81.0%), (c) 100.0% (100.0–100.0%) vs. 97.5% (94.2–100.0%), and (d) 93.6% (87.4–100.0%) vs. 74.9% (67.9–82.6%).
